# Obituary

**Published:** 1879-09

**Authors:** 


### OBITUARY.

Died at the Sherman House, in Chicago, on the 10th of August, Dr. A. M. Moore, of Lafayette, Ind.

Dr. Moore was a prominent citizen of that city, was engaged in the successful practice of dentistry for many years.

He stood well in the profession of which he was an honored member. He was for one year professor of clinical dentistry, in the Ohio College of Dental Surgery. Till within the past five or six years, he had been quite successful in business. But the financial depression that every where prevailed, affected him seriously, sweeping away most of his accumulations, and occasioning much mental depression, against this he struggled heroically. With the view of retrieving his losses, he entered upon some speculations that resulted disastrously, and added greatly to his trouble. On the evening before his death, it was noticed that he was laboring under great depression, and his general bearing that of a man who was loaded down with mental and physical disability. An employe of the house assisted him to his room, where he expired, it is supposed, a few hours afterwards. He was about fifty years of age.

